# Unheard voices of global surgery – trauma services in sub-Saharan Africa: a correspondence

**DOI:** 10.1097/JS9.0000000000000145

**Published:** 2023-03-02

**Authors:** Aashna Mehta, Andrew A. Wireko, Pearl Ohenewaa Tenkorang, Jyi Cheng Ng, Toufik-Abdul Rahman, Vladyslav Sikora, Arda Isik

**Affiliations:** a Faculty of Medicine, University of Debrecen, Debrecen, Hungary; b Sumy State University, Sumy, Ukraine; c University of Ghana Medical School, Accra, Ghana; d Faculty of Medicine and Health Sciences, University of Putra Malaysia, Serdang, Malaysia; e Department of General Surgery, Istanbul Medeniyet University, Istanbul, Turkey

Dear Editor,

According to the 2015 Global Burden of Disease (GBD) study, an estimated 4.8 million people die as a result of trauma each year, accounting for 8.5% of global deaths^1^. As a result, it is estimated that more than 90% of these deaths occur in low-income and middle-income countries^2^. Road traffic injuries account for ~7.7% of all disability-adjusted life years, with sub-Saharan Africa (SSA) having the highest rate of accident-related mortality^2^. For example, in 2019, Ethiopia, Uganda, Zimbabwe, and South Africa had the highest proportion of injury-related deaths^2^. At least one-third of these deaths are attributable to a lack of access to trauma surgery, which results in avoidable mortality, morbidity, and lost productivity in the region, perpetuating the vicious cycle of socioeconomic inequity^3^.

Since Farmer and Kim^4^ described surgery in 2008 as a “neglected stepchild of global public health,” global surgery has become a field of immense interest and landmark research. Although SSA bears the brunt of trauma cases, there has been significant progress in trauma case management in recent years. This can be seen in the establishment of a partnership program between trauma care instructors from the United Kingdom and trauma care trainees from nine different SSA countries^5^. The program was called COSECSA-Oxford-Orthopedic-Link (COOL) which trained 540 doctors, 260 nurses, 119 clinical officers, and 111 medical students in primary trauma care in SSA^5^. Furthermore, the World Health Organization (WHO), in collaboration with the International Society of Surgery (ISS) and the International Association for Trauma Surgery and Intensive Care (IATSIC), has made significant efforts to develop quality improvement programs for better trauma management, particularly in low-resource settings^6^.

Despite widespread recognition and efforts, trauma services in SSA continue to be underrepresented on the international stage, as their needs go unmet^7^. There is little research on trauma surgery in SSA resulting in a scarcity of data in the field to critically assess its needs. More so, SSA had the lowest authorship contribution per article in collaboration on surgical research, with trauma surgery being the least represented specialty within global surgery^7^,^8^. While disparities with high-income countries are acknowledged, internal disparities should also be noted. In SSA, trauma care is distributed inequitably. Access to high-quality trauma care is readily available in urban areas and among populations with a higher socioeconomic status, whereas rural and lower-class regions have limited or no access despite a higher demand for trauma care in these rural areas^2^. These disparities are caused by differences due to socioeconomic status, sex, age, physical disability, sexual orientation, and geographic location^2^. The underdeveloped trauma care management in SSA is primarily due to a lack of infrastructure and a skilled health workforce. In addition, limited trauma care training and education, insufficient funds and poor health insurance schemes, a lack of necessary logistics and equipment, and a high brain drain of trauma care personnel are all factors impeding trauma care progress in SSA^2^.

In view of the foregoing, it is crucial to imply strategies to improve trauma care services in SSA. Of note, is the need for improved investment in updating the current health infrastructure as the limited availability of trauma personnel and resources often impedes access to life-saving care. SSA could consider establishing specialized trauma centers that offer comprehensive care to trauma patients. Besides, training the primary care team to manage basic trauma cases can considerably help reduce morbidity and mortality from injuries and trauma in SSA, especially in areas with limited availability of trauma surgeons. Establishing international partnerships and collaborations with trauma centers worldwide can further improve visibility and open opportunities for educational and vocational training. These measures can considerably improve trauma workforce capacity, a step towards standardized trauma care delivery in SSA.

In addition, regular quality monitoring audits can help identify relevant (international, national, and local) issues faced in everyday trauma practice, as well as guide potential solutions to improve care provision locally. Results obtained by these audits and quality improvement surveys can promote public health policy reform and ultimately up-to-date clinical practice guidelines that address the current trauma needs of the SSA region. Therefore, identifying and addressing the unique challenges faced in trauma care provision is of paramount significance in reducing morbidity and mortality from otherwise treatable injuries in the SSA region.

## Ethical Approval and Consent to Participate

Not applicable

## Funding

None.

## Authors’ Contributions

A.M.: Conceptualization ideas: All authors were involved in data curation, writing of initial draft, review and editing, final draft.

## Conflicts of Interest Statement

Authors declare no conflict of interest.

## Consent for publication

Not applicable.

## Availability of supporting data

No new data generated.

## Research Registration Unique Identifying Number (UIN)

None.

## Conflicts of interest

The authors declare that they have no financial conflict of interest with regard to the content of this report.

## Guarantor

Wireko Andrew Awuah.
